# Influence of Intervertebral Fixation and Segmental Thrust Level on Immediate Post-Spinal Manipulation Trunk Muscle Spindle Response in an Animal Model

**DOI:** 10.3390/brainsci11081022

**Published:** 2021-07-31

**Authors:** Carla R. Lima, Daniel F. Martins, Snigdhasree Avatapally, Minjung Cho, Peng Li, William R. Reed

**Affiliations:** 1 Rehabilitation Science Program, University of Alabama at Birmingham, Birmingham, AL 35294, USA; clima@uab.edu; 2 Experimental Neuroscience Laboratory (LaNEx), Postgraduate Program in Health Sciences, University of Southern Santa Catarina, Palhoça, SC 88137-270, Brazil; daniel.martins4@unisul.br; 3 Research Honors Undergraduate Program, University of Alabama at Birmingham, Birmingham, AL 35294, USA; snigdha3@uab.edu (S.A.); mincho22@uab.edu (M.C.); 4 School of Nursing, University of Alabama at Birmingham, Birmingham, AL 35294, USA; pli@uab.edu; 5 Department of Physical Therapy, School of Health Professions, University of Alabama at Birmingham, Birmingham, AL 35294, USA

**Keywords:** muscle spindle, spinal manipulation, trunk, hypomobility, facet joint, cat, spine

## Abstract

Objective: To characterize the effect of unilateral (single and two-level) lumbar facet/zygapophysial joint fixation on paraspinal muscle spindle activity immediately following L4 or L6 high velocity low amplitude spinal manipulation (HVLA-SM) delivered at various thrust durations. Methods: Secondary analysis of immediate (≤2 s) post-HVLA-SM trunk muscle spindle response from two studies involving anesthetized adult cats (*n* = 39; 2.3–6.0 kg) with either a unilateral single (L5/6) or two-level (L5/6 and L6/7) facet joint fixation. All facet fixations were contralateral to L6 dorsal root recordings. HVLA-SM was delivered to the spinous process in a posterior-to-anterior direction using a feedback motor with a peak thrust magnitude of 55% of average cat body weight and thrust durations of 75, 100, 150, and 250 ms. Time to 1st action potential and spindle activity during 1 and 2 s post-HVLA-SM comparisons were made between facet joint fixation conditions and HVLA-SM segmental thrust levels. Results: Neither two-level facet joint fixation, nor HVLA-SM segmental level significantly altered immediate post-HVLA-SM spindle discharge at tested thrust durations (FDR > 0.05). Conclusions: Two-level facet joint fixation failed to alter immediate (≤2 s) post-HVLA-SM spindle discharge when compared to single-level facet joint fixation at any thrust duration. Segmental thrust level did not alter immediate post-HVLA-SM spindle response in two-level facet joint fixation preparations.

## 1. Introduction

Muscle-related abnormalities such as co-contraction, loss of precise motor trunk control, and diminished trunk proprioception are considered likely contributors to LBP onset, severity, and/or chronicity [1,2,3]. Understanding how pathophysiological trunk/spinal conditions such as spinal joint hypomobility, facet joint hypertrophy, and disc degeneration influence trunk proprioception and/or alter trunk motor control becomes crucial to finding effective evidence-based clinical treatments for chronic LBP. Recent studies using quantitative fluoroscopy suggest that motion sharing inequality between spinal segments (i.e., aberrant sharing of motion between each segment) due to muscle hypertonicity, and hypo- and/or hyper-intervertebral joint mobility is significantly higher in individuals with LBP when compared to asymptomatic controls [4,5]. This segmental motion sharing inequality was present during passive and active trunk flexion, extension and lateral flexion motions in individuals with LBP [4], and could contribute to proprioceptive feedback errors arising from muscle spindle afferents in small intervertebral or large paraspinal muscles.

Muscle spindles are mechanoreceptors responsible for detecting changes in muscle length and the velocity of such length changes among extrafusal muscle fibers [6,7,8]. In animal models, painful musculoskeletal conditions have been shown to increase γ-motor neuron sensitivity and muscle spindle response to subsequent stretches consequently leading to α-motor neuron hyperexcitation [9,10,11,12,13,14]. However, little is known regarding the immediate and long-term effects of reduced intervertebral mobility on trunk proprioception or trunk muscle spindle response to applied spinal forces such as those delivered during high-velocity low-amplitude spinal manipulation (HVLA-SM). The effects of physical characteristics of HVLA-SM thrust delivery (i.e., thrust magnitude, thrust duration) in unilateral single-(L5/6) and multi-level (L5/6, L6/7) facet-joint fixation feline preparations was recently investigated [15,16]. Unilateral facet joint fixations increased spinal joint stiffness (i.e., decreased intervertebral mobility) and reduced muscle spindle discharge during the actual HVLA-SM thrust itself (baseline to peak force), particularly at thrust durations shorter than 150 ms [15,16]. In addition, this work demonstrated for the first time the effect of remotely delivered HVLA-SM (at L4) on L6 trunk muscle spindle response with and without unilateral facet joint fixation [16]. Whereas the L6 muscle spindle response decreased with L4 HVLA-SM compared to L6 HVLA-SM, 60% to 80% of an L6 HVLA-SM muscle spindle response was still elicited from a remotely delivered L4 HVLA-SM both in the absence and presence of facet joint fixation [16]. This study demonstrated for the first time a regional HVLA-SM mechanoreceptor activation gradient related to the propagation of applied HVLA-SM forces. However, these intervertebral joint fixation muscle spindle studies failed to characterize the effects of unilateral single and two-level facet joint fixation on L6 muscle spindle response immediately (≤2 s) following the delivery of L4 or L6 HVLA-SM. Therefore, the purpose of this secondary analysis was to characterize the immediate (≤2 s) post-HVLA-SM trunk muscle spindle response in unilateral single- and two-level facet- joint fixated feline preparations at different HVLA-SM thrust durations and/or segmental HVLA-SM thrust levels. We hypothesize that compared to unilateral single-level facet joint fixation (L5/6), two-level facet-joint fixation (L5/L6, L6/L7) will significantly decrease post-HVLA-SM’s time to 1st action potential (AP; seconds) and increase spindle discharge during 1 and 2 s post-HVLA-SM due to decreased HVLA-SM related intervertebral motion and an increased recovery to muscle spindle resting discharge. In addition, we hypothesize that an HVLA-SM thrust delivered at L4 will decrease post-HVLA-SM’s time to 1st AP and increase spindle activity during 1 s and 2 s compared to L6 delivered HVLA-SM thrusts in two-level (L5/6, L6/7) facet-joint fixated preparations due to a resultant decrease in the overall propagation of intervertebral motion arising from the L4 HVLA-SM and a more rapid return to muscle spindle resting discharge.

## 2. Materials and Methods

**Figure 1:**
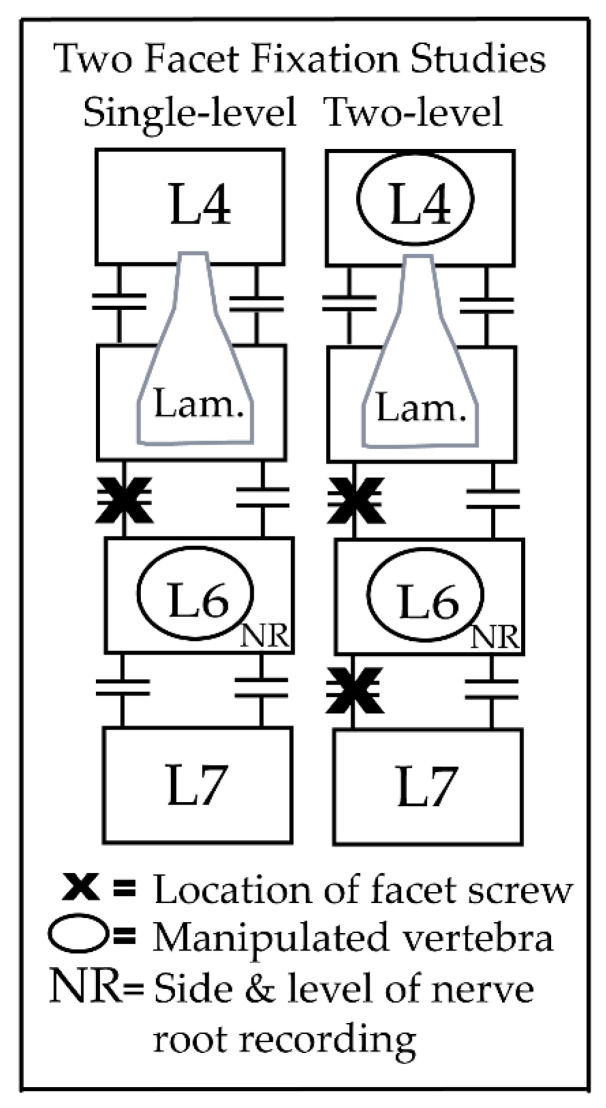
Diagram of single and two-level facet joint fixation experimental preparation.

This study is a secondary analysis of data collected using a single and two-level facet joint fixation in adult male feline preparations [15,16,17]. All experiments were approved by the Institutional Animal Care and Use Committee of Palmer College of Chiropractic. Animals (*n* = 39; 2.3–6.0 kg) were deeply anesthetized and L6 dorsal root trunk muscle spindle activity was recorded following simulated L4 or L6 HVLA-SM delivery. All general surgical and electrophysiological procedures have been previously described in greater detail elsewhere [15,16,17]. Briefly, anesthesia was introduced with isoflurane and maintained with Nembutal (35 mg/kg, i.v.; Oak Pharmaceuticals, Lake Forest, IL, USA). Catheters were placed in the carotid artery and external jugular vein for blood pressure monitoring and fluid introduction. A tracheal tube was inserted allowing artificial ventilation and blood oxygenation parameters were maintained within the normal range (PCO_2_ = 32–37 mmHg; PO_2_, > 85 mmHg; and pH = 7.32–7.43). As we were interested in trunk muscle spindle afferent responses to HVLA-SM, hindlimb afferent input was reduced by cutting the right sciatic nerve. For the electrophysiological recordings, a laminectomy at the L5 level was performed (including removal of the L5 spinous process) exposing the L6 dorsal rootlets (Figure 1). Teased dorsal rootlets were then placed on a monopolar electrode platform. To induce spinal joint hypomobility, titanium endosteally anchored miniscrews (10 mm tomas-pin; Dentaurum, Ispringen, Germany) were unilaterally placed in either the left L5/6 facet joints (single-level) or at the left L5/6 and L6/7 facet joints (two- level) (Figure 1; for experimental preparation X-rays and photographs, see [15,16]). The paraspinal muscles on the right side remained intact and L6 muscle spindle receptive fields were located in the multifidus or longissimus muscles. This experimental preparation of unilateral single and two-level facet joint fixation was designed to reduce overall L6 segmental mobility (as commonly reported clinically) while not disrupting the contralateral paraspinal muscle tissue or adversely impacting L4 segmental mobility. All spindle afferents increased their mean discharge frequency after succinylcholine injection (100 mg/kg; Butler Schein, OH), sustained responses to fast vibratory stimuli (~70 Hz), and decreased discharge to muscle twitch caused by bipolar direct muscle stimulation (0.2–0.3 mA; 50 µs).

### 2.1. HVLA-SM

Simulated HVLA-SM was delivered directly onto either the L4 or L6 spinous process in the posterior-anterior (P-A) direction using a computer-controlled feedback motor control system (Aurora Scientific, Lever System Model 310) under force control (which increased at a constant rate until peak force was obtained). A greater description of the feedback motor, its calibration and simulated HVLA-SM delivery is reported elsewhere [18]. HVLA-SM thrust magnitude remained constant at 55% body weight (BW) based on the average cat body weight of 3.95 kg [18,19]. HVLA-SM thrust durations of 0 (non-thrust control), 75, 100, 150, and 250 ms were delivered randomly to prevent ordering effects. HVLA-SM were delivered via forceps directly attached to the exposed L4 or L6 spinous process. As fixation data were analyzed from two separate fixation studies, only the two-level facet joint fixation preparations received HVLA-SM delivered at both L4 and L6, whereas the single-level fixated preparations received only L6 HVLA-SM. A period of 5 min elapsed between HVLA-SM thrusts to allow adequate time for recovery of viscoelastic properties of paraspinal soft tissues [19].

### 2.2. Statistical Analysis

**Figure 2:**
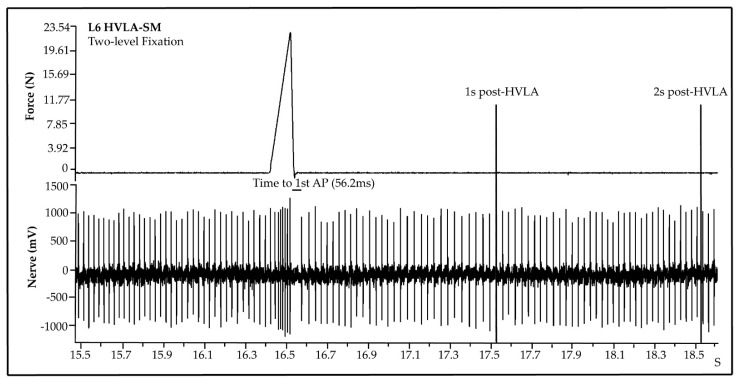
Post-HVLA-SM outcomes. A representative example of a raw muscle spindle recording of a simulated L6 HVLA-SM with a 100 ms thrust duration. Measured outcomes include time to 1st action potential (AP), and frequency discharge during 1 and 2 s post-HVLA-SM.

L6 dorsal root muscle spindle discharge was recorded prior to and at 2 s following HVLA-SM thrust delivery in single- or two-level facet joint fixation preparations. The normality assumption was checked using Q–Q plots and all data are presented as mean ± standard deviation (SD). A Wilcoxon rank sum test was conducted to examine the effects of single and two-level facet joint fixation and segmental thrust location on each outcome of interest (i.e., time to 1st AP, muscle spindle activity during 1 and 2 s; see Figure 2) at varied thrust durations. Due to the large number of comparisons, the false discovery rate (FDR) was used for the multiple comparison correction with FDR values < 0.05 being considered as statistically significant. All the analyses were conducted using SAS 9.4 (Cary, NC, USA).

## 3. Results

Unilateral two-level facet joint fixation (L5/6 and L6/7) failed to significantly alter immediate (≤2 s) post-HVLA-SM muscle spindle discharge when compared to single-level (L5/6) facet joint fixation at any non-control HVLA-SM thrust duration (FDR > 0.05; Table 1). There were also no differences between L4 and L6 delivered HVLA-SM in immediate post-HVLA-SM spindle discharge in the two-level facet joint fixation preparations at any non-control thrust duration (FDR > 0.05; Table 2).

## 4. Discussion

In this study, we conducted a secondary analysis of immediate (≤2 s) post-HVLA-SM muscle spindle activity in feline preparations with intervertebral motion restriction induced by fixating either single-level (left L5/6) or two-level (left L5/6, L6/7) facet joints [15,16]. We found that two-level facet joint fixation failed to significantly alter immediate post-HVLA-SM spindle activity (time to 1st AP, and discharge activity during 1 and 2 s) at any non-control thrust duration when compared to responses in single- level facet joint fixated preparations. In addition, no differences in immediate (≤2 s) post-HVLA spindle activity were found between L4- and L6- delivered HVLA-SM. Responses to L7 HVLA-SM thrusts were not included in these fixation studies; however, muscle spindle response comparisons between L6 and L7 HVLA-SM have been reported in a separate non-fixated feline study [20].

In previous work we demonstrated that compared to a laminectomy-only condition, single and two-level unilateral facet joint fixation increased L6 spinal stiffness and decreased muscle spindle discharge during the HVLA-SM thrust delivery (baseline to peak force) itself, particularly during shorter HVLA-SM thrust durations (≤150 ms) [15,16]. Mild spinal hypomobility (i.e., unilateral facet joint fixation) appears to alter muscle spindle discharge only during the delivery of the HVLA-SM thrust itself and not immediately (≤2 s) afterwards, as no significant differences were found in immediate post-HVLA-SM response between single-level and two-level facet joint fixated preparations at any non-control HVLA-SM thrust duration (Table 1). Similar thrust duration results were also reported in a recent study investigating the effects of HVLA-SM thrust duration (25, 50, 75, 100, 150, 200, and 250 ms) and thrust magnitude (25%, 55%, 85% cat (BW)) on immediate (≤2 s) post-HVLA-SM spindle discharge in non-fixated preparations [21]. In non-fixated feline preparations, while no significant differences between HVLA-SM thrust durations (25–250 ms) and immediate (≤2 s) post-HVLA-SM muscle spindle responses were found, we did find that 55% BW thrust magnitude increased post-HVLA-SM time to 1st AP and decreased spindle discharge during both 1 and 2 s compared to control (non-thrust) [21]. It should also be noted that this unilateral facet joint fixation model was specifically selected opposed to more aggressive intervertebral cages or steel rod fixation as unilateral facet fixation provides only partial (not complete) intersegmental hypomobility which is more translationally relevant for clinical spinal manipulation treatment which is rarely delivered to completely immobile/fused vertebra segments. While not translationally relevant, complete intervertebral joint fixation or fusion may alter immediate (≤2 s) post-HVLA-SM muscle spindle responses in different ways than the unilateral facet joint fixation model used in this study. Performance of bilateral or right-only facet joint fixation in this experimental preparation would prove difficult without disrupting the right lumbar paraspinal muscle.

In the current study, we failed to find any significant differences in any of the three immediate post-HVLA-SM outcomes measured (time to 1st AP, and discharge activity during 1 and 2 s) comparing L4 vs. L6 HVLA-SM in two-level preparations. The lack of HVLA-SM thrust durations altering immediate (≤2 s) post-HVLA-SM response in either non-fixated [21] or facet joint fixated preparations suggests that factors inherent to the muscle spindle apparatus likely dictate immediate HVLA-SM muscle spindle response recovery and that HVLA-SM physical characteristics other than thrust duration (i.e., thrust magnitude) may be of greater importance to post-HVLA-SM spindle response.

Future studies investigating immediate post-HVLA-SM muscle spindle response to extremely short durations (2–5 ms) as delivered by commercially available clinical HVLA-SM devices (i.e., Activator^®^, Pulstar^®^, Impulse^®^ instruments) may help to clarify the presence of any inherent physiological limitations to immediate HVLA-SM muscle spindle recovery response. Studies with longer post-HVLA-SM periods are needed. In addition, the influence of pro-inflammatory and/or other chronic nociceptive mediators, as well as the influence of chronic intervertebral disc injury/degeneration on trunk muscle spindle response to muscle stretch and/or spinal manipulation, need to be investigated to provide a more complete and mechanistic understanding of the neurophysiological response to HVLA-SM.

## 5. Limitations

There are several limitations associated with this study, including the following: (1) only immediate (≤2 s) post-HVLA-SM muscle spindle response were evaluated, longer post-HVLA-SM periods (i.e., minutes) should be investigated; (2) HVLA-SM was not delivered at the end-range of vertebral motion as performed clinically to minimize possible nerve fiber damage or tearing from the recording electrode; (3) simulated HVLA-SM was directed in a posterior-to-anterior direction only, while clinically delivered HVLA-SM is typically delivered with superior-to-inferior and medial-to-lateral components depending on the segmental level and plane line of the intervertebral disc; (4) post-HVLA-SM muscle spindle responses were recorded in the absence of any muscle tissue injury or inflammation which typically accompanies clinical LBP; (5) the influence of extremely short HVLA-SM thrust durations (2–5 ms) associated with clinical HVLA-SM mechanical devices on immediate post-HVLA-SM spindle response was not evaluated, and (6) segmental stiffness in feline lumbar spines tends to be up to 7× lower than that found in humans equating to greater vertebral translation or intervertebral motion during HVLA-SM in spite of unilateral facet joint fixation.

## 6. Conclusions

In conclusion, no differences in immediate (≤2 s) post-HVLA-SM response between single and two-level facet joint fixation preparations at different HVLA-SM thrust durations (75–250 ms) were found. In addition, segmental thrust location (L4, L6) failed to alter immediate (<2 s) post-HVLA-SM response providing additional evidence that precise segmental HVLA-SM thrust accuracy may not be essential to achieve clinical efficacy [16,22,23], if indeed muscle spindle response is later determined (as long theorized [24,25,26]) to be an important factor to the clinical efficacy of spinal manipulation. Collectively, these findings suggest that mild intervertebral joint hypomobility fails to alter immediate post-HVLA-SM response and suggests that immediate post-HVLA-SM muscle spindle response may be inherently limited physiologically following manually delivered relevant HVLA-SM thrust durations of 75–250 ms.

## Figures and Tables

**Table 1:** Thrust duration comparisons between 1 and 2 screws.

Outcomes	Thrust Magnitude (%BW)	Thrust Duration (ms)	# of Screws	*n*	Mean	SD	*p*	FDR
Time to 1st AP (s)	0%	0	1	18	0.035	0.020	0.0481 *	0.5115
			2	21	0.023	0.013		
	55%	75	1	18	0.089	0.065	0.4433	0.5115
			2	21	0.105	0.070		
	55%	100	1	18	0.097	0.065	0.3103	0.5115
			2	21	0.118	0.074		
	55%	150	1	18	0.106	0.079	0.3956	0.5115
			2	21	0.123	0.084		
	55%	250	1	18	0.129	0.103	0.7058	0.7562
			2	21	0.155	0.162		
1s post-thrust (Hz)	0%	0	1	18	34.00	9.28	0.2606	0.5115
			2	21	31.10	13.54		
	55%	75	1	18	28.44	11.46	0.3365	0.5115
			2	21	25.14	13.37		
	55%	100	1	18	27.44	10.73	0.1707	0.5115
			2	21	24.24	13.36		
	55%	150	1	18	27.00	11.08	0.3506	0.5115
			2	21	24.38	13.04		
	55%	250	1	18	24.89	11.98	0.7582	0.7582
			2	21	23.57	13.33		
2 s post-thrust (Hz)	0%	0	1	18	68.89	18.51	0.2847	0.5115
			2	21	62.67	26.66		
	55%	75	1	18	59.94	21.59	0.2608	0.5115
			2	21	53.10	26.42		
	55%	100	1	18	58.11	20.30	0.2495	0.5115
			2	21	52.24	27.00		
	55%	150	1	18	58.83	20.82	0.3581	0.5115
			2	21	53.38	26.34		
	55%	250	1	18	55.28	21.92	0.4348	0.5115
			2	21	51.10	26.81		

Note: %BW = % of body weight; ms = milliseconds; s = seconds; AP = action potential; Hz = hertz; *n* = sample; M = mean; SD = standard deviation; * = *p* < 0.05; FDR = False discovery rate.

**Table 2:** Thrust Duration Comparisons Between L4 and L6 HVLA-SM.

Outcomes	Thrust Magnitude (%BW)	Thrust Duration (ms)	# of Screws	Vertebra	*n*	Mean	SD	*p*
Time to 1st AP (s)	0%	0	2	L4	20	0.020	0.013	0.4462
				L6	21	0.023	0.013	
	55%	75	2	L4	20	0.081	0.065	0.2323
				L6	21	0.105	0.070	
	55%	100	2	L4	20	0.090	0.061	0.2226
				L6	21	0.118	0.074	
	55%	150	2	L4	19	0.105	0.068	0.6097
				L6	21	0.123	0.084	
	55%	250	2	L4	19	0.116	0.086	0.5728
				L6	21	0.155	0.162	
1 s post-thrust (Hz)	0%	0	2	L4	20	30.80	11.55	0.9483
				L6	21	31.10	13.54	
	55%	75	2	L4	20	26.70	11.96	0.6595
				L6	21	25.14	13.37	
	55%	100	2	L4	20	26.00	11.78	0.4454
				L6	21	24.24	13.36	
	55%	150	2	L4	20	25.80	12.80	0.5775
				L6	21	24.38	13.04	
	55%	250	2	L4	20	24.95	11.90	0.5952
				L6	21	23.57	13.33	
2 s post-thrust (Hz)	0%	0	2	L4	20	61.95	23.02	0.9999
				L6	21	62.67	26.66	
	55%	75	2	L4	20	55.20	22.94	0.6503
				L6	21	53.10	26.42	
	55%	100	2	L4	20	55.20	22.98	0.4851
				L6	21	52.24	27.00	
	55%	150	2	L4	20	54.05	23.88	0.7557
				L6	21	53.38	26.34	
	55%	250	2	L4	20	52.60	23.15	0.6690
				L6	21	51.10	26.81	

Note: %BW = % of body weight; ms = milliseconds; s = seconds; AP= action potential; Hz = Hertz; *n* = sample; M = mean; SD = standard deviation; FDR = False Discovery Rate. In certain experiments, the neural recording was lost prior to both L4 and L6 manipulative protocol completion accounting for the small differences in sample size between thrusts delivered at both spinal locations.

## Data Availability

Data are available upon request from the corresponding author. The data are not publicly available due to electronic security concerns.
